# The carnitine status does not affect the contractile and metabolic phenotype of skeletal muscle in pigs

**DOI:** 10.1186/s12986-017-0238-7

**Published:** 2018-01-10

**Authors:** Daniel Kaup, Janine Keller, Erika Most, Joachim Geyer, Klaus Eder, Robert Ringseis

**Affiliations:** 1 Institute of Animal Nutrition and Nutrition Physiology, Justus-Liebig-University of Giessen, Heinrich-Buff-Ring 26-32, 35392, Giessen, Germany; 2 Institute of Pharmacology and Toxicology, Justus-Liebig-University of Giessen, Schubertstr 81, 35392, Giessen, Germany

**Keywords:** Carnitine, Muscle fiber distribution, Pig, Myosin heavy chain isoforms

## Abstract

**Background:**

Recently, supplementation of L-carnitine to obese rats was found to improve the carnitine status and to counteract an obesity-induced muscle fiber transition from type I to type II. However, it has not been resolved if the change of muscle fiber distribution induced in obese rats and the restoration of the “normal” muscle fiber distribution, which is found in lean rats, in obese rats by supplemental L-carnitine is causally linked with the carnitine status. In the present study we hypothesized that fiber type distribution in skeletal muscle is dependent on carnitine status.

**Methods:**

To test this, an experiment with 48 piglets which were randomly allocated to four groups (*n* = 12) was performed. All piglets were given orally either 60 mg sodium bicarbonate/kg body weight (group CON), 20 mg L-carnitine and 60 mg sodium bicarbonate/kg body weight (group CARN), 30 mg pivalate (dissolved in sodium bicarbonate)/kg body weight (group PIV) or 20 mg L-carnitine and 30 mg pivalate/kg body weight (group CARN + PIV) each day for a period of 4 weeks.

**Results:**

Concentrations of total carnitine in plasma, liver and longissimus dorsi and biceps femoris muscles were 2.0–2.7 fold higher in group CARN than in group CON, whereas these concentrations were 1.9–2.5-fold lower in group PIV than in group CON. The concentrations of total carnitine in these tissues did not statistically differ between group CARN + PIV and group CON. Fiber type distribution of longissimus dorsi and biceps femoris muscles, mRNA and protein levels of molecular regulators of fiber distribution in longissimus dorsi and biceps femoris muscles and mRNA levels of genes reflecting the metabolic phenotype of longissimus dorsi and biceps femoris muscles did not differ between groups.

**Conclusion:**

Changes in the systemic carnitine status and the muscle carnitine concentration induced by either supplementing L-carnitine or administering pivalate have no impact on the contractile and metabolic phenotype of skeletal muscles in pigs.

## Background

Individual skeletal muscles in the body have distinct contractile and metabolic functions depending on their location in the body and the kind of muscular effort. The anatomic basis for the distinct functions of individual skeletal muscles is their specific distribution pattern of muscle fibers, from which two major types differing in their myosin heavy chain (MHC) isoforms and their enzymatic capacity [1] can be distinguished. It has long been known that the oxidative type I fibers expressing MHC I are mitochondria-rich, have a high oxidative capacity utilizing mostly oxidative phosphorylation for energy production, while the glycolytic type II fibers, which are subdivided into IIA expressing MHC IIa, IIb expressing MHC IIb and IIx expressing MHC IIx, have a lower oxidative capacity due to fewer mitochondria content than type I muscle fibers and thus depend on glycolytic metabolism to generate ATP [2]. According to recent evidence, the different energy substrate preferences between the two major types of muscle fibers are not only explained by the different number of mitochondria but also by mitochondrial specialization across fiber types with regard to regulation of oxidative phosphorylation, dynamics of reactive oxygen species, handling of Ca^2+^ and regulation of cell death [3].

Noteworthy, the fiber distribution of a given muscles shows high plasticity and can be induced to switch depending on various factors, like exercise, mechanical unloading and obesity, thereby leading to a change of the muscle’s metabolic phenotype [4–7]. With regard to obesity, it has been shown that obese subjects have a lower percentage of type I and a higher percentage of type II fibers and an impaired mitochondrial oxidative enzyme capacity in skeletal muscle than lean counterparts [7, 8] indicating that type I fibers are initiated to switch into type II fibers during obesity development. Interestingly, we have found recently that supplementation of L-carnitine to obese rats counteracts the obesity-induced muscle fiber transition from type I to type II through inducing genes encoding critical molecular regulators of muscle fiber transition, like peroxisome proliferator-activated receptor δ (PPARδ encoded by PPARD), PPARγ coactivator-1α (PGC-1α encoded by PPARGC1A) and PGC-1β (PPARGC1B) [9–12], and thereby favours an oxidative metabolic phenotype of skeletal muscle [13]. L-Carnitine is a water soluble quaternary amine which is essential for normal function of all tissues and is derived from both dietary sources (meat and dairy products) and endogenous biosynthesis [14]. The most reported function of L-carnitine is its role in the import of long-chain fatty acids from the cytosol into the mitochondrial matrix for subsequent β-oxidation [15]. However, L-carnitine is generally important for proper functioning of intermediary metabolism considering that deficiency of L-carnitine is associated not only with impaired fatty acid utilization but also with perturbations of glucose utilization and insulin sensitivity [16].

We have also found in our recent study that obese rats receiving no supplemental L-carnitine had significantly lower tissue carnitine concentrations than healthy lean rats, while supplementation of L-carnitine to the obese rats increased tissue carnitine concentrations to levels found in lean rats [13]. It is unclear however if the above described change of muscle fiber distribution induced in obese rats and the restoration of the “normal” muscle fiber distribution, which is found in lean rats, in obese rats by supplemental L-carnitine is causally linked with the carnitine status. In the present study we hypothesized that the fiber type distribution in skeletal muscle is dependent on the carnitine status. To test this hypothesis we experimentally manipulated the carnitine status of young pigs as an animal model through oral administration of either L-carnitine or sodium pivalate. Administration of sodium pivalate, the pivalic acid (trimethylacetic acid) mojety of pivampicillin, is an established model of secondary carnitine deficiency [17]. In humans, secondary carnitine deficiency is known to be caused as a side effect of pharmacologic treatment with pivalate and valproate and by inborn metabolic disorders, diabetes, renal tubular loss, haemodialysis and peritoneal dialysis [18]. Pivalic acid induces carnitine deficiency in both humans and laboratory animals by forming excessive tissue levels of pivaloylcarnitine esters [19, 20], which are readily released from tissues and subsequently lost in the urine at a rate that exceeds endogenous carnitine synthesis. Young pigs were used as experimental animals, because previous studies of our group demonstrated that the carnitine concentration in skeletal muscle of young pigs can be markedly increased within a relatively short feeding period of about three weeks [21, 22]. In contrast to pigs, studies in healthy rodents (mice and rats) demonstrated that even high doses of supplemental L-carnitine cause either no [23, 24] or only a slight increase of muscle carnitine concentration [25]. In line with this, fiber type type distribution in healthy mice was not affected by supplemental carnitine [24]. Like in mice, studies in human subjects showed that supplementation of carnitine for several weeks does not alter the muscle free and/or total carnitine content [26–28] and the fiber composition of vastus lateralis muscle, a muscle with a balanced type I and type II fiber distribution, [28, 29]. Following treatment of pigs for four weeks, carnitine status was assessed by HPLC-MS/MS and muscle fiber typing was performed using MHC distribution-based immunohistochemistry. In addition, the expression of genes reflecting the metabolic phenotype and genes encoding critical regulators of muscle fiber transition were determined in skeletal muscle. In order to exclude the possibility that sodium pivalate exerts an effect on muscle fiber distribution independent of its effect on carnitine status we also considered pigs that received orally sodium pivalate and L-carnitine.

## Methods

### Animals and diets

The animal experiments were approved by the local Animal Care and Use Committee (Regierungspräsidium Giessen; permission no: GI 19/3 No. 30/2013). All experimental procedures described followed established guidelines for the care and handling of laboratory animals. Two experiments with 5 weeks old, male crossbred piglets [Pietrain x (German Landrace x German Edelschwein)] were carried out. In both experiments, the piglets were kept in pairs in flat-deck pens under controlled conditions (23 ± 2 °C room temperature, 50–60% relative humidity, light from 06.00 a.m. to 07.00 p.m.), and all groups received an identical diet which met the nutrient requirements according to German Society for Nutrition Physiology [30]. According to a phase feeding system, the piglets received two diets, which differed in the protein:energy ratio, throughout the experiment: one from the start of the experiment until a of body weight of 15 kg (phase I), and a second one until the end of the experiments at day 28 (phase II). The diet in phase I consisted of (g/kg): wheat, 381.7; barley, 315; soybean meal (44% CP), 250; soybean oil, 15; mineral and vitamin premix, 33.5; L-lysin, 2.6; DL-methionine, 1.0 and L-threonine, 1.2. In phase II the diet consisted of (g/kg): wheat, 401.9; barley, 302; soybean meal (44% CP), 240; soybean oil, 15; mineral and vitamin premix, 33.4; L-lysin, 1.5; DL-methionine, 0.5 and L-threonine, 0.7 and a mixture of organic acids (containing 45% formic acid, 7.7% lactic acid, 5% sodium acetate), 5. The carnitine concentration in the diets was below 10 mg/kg diet as analyzed by tandem mass spectrometry according to Hirche et al. [31]. Water was constantly available ad libitum from nipple drinkers during both experiments.

In experiment 1, which aimed to determine the effective pivalate dose, piglets were randomly allocated to five groups of 4 piglets each. At each day of the 28 days period, the piglets were given orally either 60 mg NaHCO_3_/kg body weight (BW) (group CON), 20 mg L-carnitine and 60 mg NaHCO_3_/kg BW (group CARN), 30 mg pivalate (dissolved in NaHCO_3_)/kg BW (group PIV30), 60 mg pivalate (dissolved in NaHCO_3_)/kg BW (group PIV60) or 120 mg pivalate (dissolved in NaHCO_3_)/kg BW (group PIV120). The carnitine dose applied is roughly comparable to that dose ingested by a piglet consuming daily 770 g of a diet containing 500 mg carnitine/kg. A recent study of our group with growing pigs showed that a carnitine dose of 500 mg/kg diet causes a 2–3-fold increase of plasma and tissue total carnitine concentrations [21]. The pivalate doses tested were based on an earlier study, in which the efficacy of different pivalate doses on tissue carnitine concentrations was investigated in rats [32].

In experiment 2, which aimed to study the effect of carnitine supplementation and carnitine deficiency on muscle fiber composition, piglets were randomly allocated to four groups of 12 piglets each (CON, CARN, PIV, CARN + PIV). Piglets of groups CON, CARN and PIV were treated identically as in experiment 1 for the same 28 days period. Group PIV was identical with group PIV30 of experiment 1, because the pivalate dose 30 mg pivalate/kg BW was found to be the most effective in inducing carnitine deficiency. Piglets of group CARN + PIV were given orally 20 mg L-carnitine and 30 mg pivalate (dissolved in NaHCO_3_)/kg BW each day throughout the 28 days period.

### Sample collection

At day 29, the pigs of both experiments were killed in the post-prandial period (2 h after their last meal) by electronarcosis followed by exsanguination. Blood was collected into heparinized polyethylene tubes (Sarstedt, Nümbrecht, Germany) and plasma obtained by centrifugation at 1100 x g for 10 min at 4 °C and subsequently stored at −80 °C. Samples from longissimus dorsi (LD) and superficial biceps femoris (BF) muscles were immediately collected into 2 ml reaction tubes. After opening the abdominal cavity, the entire liver was collected and a small aliquot taken into a 2 ml reaction tube. Subsequently, all tissue samples were snap-frozen in liquid nitrogen and stored at −80 °C pending analysis.

### Carnitine analysis

Tandem mass spectrometry was used for determining the concentrations of free carnitine and acetyl-carnitine in plasma, liver and LD and BF muscles according the method of Hirche et al. [31]. Deuterated L-carnitine-methyl-d3-hydrochloride and deuterated O-acetyl-d3-carnitine-hydrochloride (Sigma-Aldrich, Steinheim, Germany) were used as internal standards.

### Fiber typing of LD and BF muscle

Frozen muscle samples from all pigs were embedded in Tissue Tek optimal cutting temperature (O.C.T.) compound (Sakura Finetek Germany GmbH, Staufen, Germany), frozen in liquid nitrogen and stored at −80 °C until cryosectioning. O.C.T.-embedded muscle samples were cut into serial 10 μm thick cross-sections with a microtome (Microm HM 500, Microm International, Walldorf, Germany) maintained at −20 °C. Fiber typing was carried out using multicolour immunofluorescence analysis according to Bloemberg and Quadrilatero [33] with following modifications: air-drying for 60 min, fixation in −20 °C methanol for 10 min prior staining, followed by a washing-step in 1×PBS and combination of blocking with the application of the first antibody cocktail. In brief, air dried and methanol fixed cross-sections were incubated with a cocktail containing primary antibodies against MHC I (BA-F8), MHC IIb (BF-F3) and all MHC isoforms except MHC IIx (BF-35) in 1xPBS containing 0.5% Triton X-100 and 10% goat serum at 4 °C overnight. Primary antibodies were purchased from Developmental Studies Hybridoma Bank (University of Iowa, USA). Following a washing step with 1× PBS, the cross-sections were incubated with different fluorescent-conjugated secondary antibodies purchased from Invitrogen (Darmstadt, Germany) in 1×PBS containing 0.5% Triton X-100 and 10% goat serum at RT for two hours. Alexa Fluor 350 IgG2b (blue) was used for BA-F8, Alexa Fluor 555 IgM (red) for BF-F3 and Alexa Fluor 488 IgG1 (green) for BF-35. Cross-sections were washed again with 1x PBS, and then mounted in Aqua Polymount (Polyscience, Niles, Illinois) and covered with coverslips. The slides were visualized with a DM 5500 B fluorescence microscope from Leica (Wetzlar, Germany) equipped with red (Excitation: 485–585 Emission: 535–685), green (Excitation: 440–520, Emission: 497–557) and blue (Excitation: 320–400, Emission: 430–510) filters, a Leica DFC340FX camera and the Leica LAS AF microscope software. Individual images were taken from the slides with each filter and a composite image was assembled automatically from the images taken with the three filters. For fiber typing, per animal all fibers within two representative fields-of-view at a 100-fold magnification were counted and typed. Blue fibers were classified as type I, red fibers as type IIb, green fibers as type IIa and fibers without staining as type IIx. For each pig a total of 729 ± 59 (mean ± SD) fibers were counted/typed in LD muscle and 777 ± 65 (mean ± SD) fibers in BF muscle.

### RNA isolation and qPCR analysis

Total RNA was isolated from 25 mg aliquots of LD and BF muscles using TRIzol reagent (Invitrogen, Karlsruhe, Germany) according to the manufacturer’s protocol. Concentration and purity of the total RNA were estimated from the optical density at 260 and 280 nm, respectively, using a NanoQuant Plate and an Infinite 200 M microplate reader (both from Tecan, Männedorf, Switzerland). The total RNA concentrations and optical density A260/A280 ratios of all LD samples were 0.496 ± 0.069 μg/μL and 1.93 ± 0.03 (mean ± SD, *n* = 48), respectively. The cDNA was synthesized by reverse transcription of 1.2 μg total RNA as described recently in detail [34]. The mRNA levels of selected genes were measured with a Rotor-Gene Q system (Qiagen, Hilden, Germany) using KAPA™ SYBR® FAST qPCR Mastermix (Peqlab, Erlangen, Germany) and gene-specific primer pairs (Eurofins MWG Operon, Ebersberg, Germany). Gene-specific primer pairs were designed using Primer3 [35] and BLAST [36]. Characteristics of primers used for qPCR analysis are shown in Table 1. The amplification of a single product of the expected size was confirmed using 2% agarose gel electrophoresis stained with GelRedTM nucleic acid gel stain (Biotium, Hayward, California, USA). Ct-values of reference and target genes were obtained using Rotor-Gene Q software (Qiagen). Ct-values were transformed into relative expression values using the 2^-ΔCt^ equation for the calculation of normalization factors. The highest relative value of each gene was set to 1. From these values, the normalization factor was calculated as the geometric mean of expression data of the three most stable out of four potential reference genes (ATP5G1, ACTB, RPS9, SHAS2) according to the method of Vandesompele et al. [37]. In LD muscle the M-values of the three most stable reference genes were: 0.498 (ATP5G1), 0.566 (ACTB) and 0.463 (RPS9). In BF muscle the M-values of the three most stable reference genes were: 0.680 (ATP5G1), 0.747 (ACTB) and 0.620 (RPS9). Ct-values of target genes were also transformed into relative expression values using the 2^-ΔCt^ equation and were normalized with the individual normalization factor resulting in relative gene quantities that were used for the statistical analysis. The mean normalized 2^-ΔCt^ ratios of the group CON was set to 1.0 and means and SD of normalized 2^-ΔCt^ ratios of the other groups (CARN, PIV, CARN + PIV) were scaled proportionally.

**Table 1:** Characteristics of gene-specific primer pairs used for qPCR

Gene	Forward primer (from 5` to 3`) Reverse primer (from 5` to 3`)	Product size (bp)	NCBI GenBank
Reference genes			
ATP5G1	CAGTCACCTTGAGCCGGGCGA TAGCGCCCCGGTGGTTTGC	94	NM_001025218
ACTB	GACATCCGCAAGGACCTCTA ACATCTGCTGGAAGGTGGAC	205	XM_003124280
RPS9	GTCGCAAGACTTATGTGACC AGCTTAAAGACCTGGGTCTG	325	XM_003356050
SHAS2	GAAAAGGCTAACCTACCCTG TGTTGGACAAGACCAGTTGG	218	NM_214053
Target genes			
ACADM	CATGGCAGCGATGTTTAGGC GGCACTTGCCCCAGAGTATT	396	NM_214039
CACT	ACTGTGACTCAGACGGACCA ATATAGCCCCCTGACACCCT	235	XM_003483178
COX4	GTGGAACTGTACCGCCTGAA TTGTCGTAGTCCCACTTGGC	257	XM_003355730
COX6	CTCAGCTCGCATGTGGAAGA GATGCGAAGATGGGGGTAGG	139	NM_001190221
FATP1	AAGGGCATGGATGATCGACT ATCACGTCTGTTGCCTGCAT	143	NM_001083931
GLUT4	TCACGTCTCTCGCTGCAGGCT AAAGCCTGTGGGGACAACACCC	205	NM_001128433
OCTN2	TGCATTTGGCTACATGCTGC ATGATCACCTCAGCTTCCTG	174	XM_003123912
PKM	CAGCAAGAAAGGTGTGAACC CTCCCTCGTGATTCTCGATT	218	XM_003356683
SDHA	CTACGCCCCCGTCGCAAAGG AGTTTGCCCCCAGGCGGTTG	380	DQ402993

### Immunoblotting

Nuclear extracts from LD and BF muscles were prepared using the Nuclear Extract Kit from Active Motif (Rixensart, Belgium) according to the manufacturer’s protocol. Protein concentrations in the nuclear extracts were determined by the bicinchoninic acid protein assay kit (Interchim, Montluçon, France) with BSA as standard. From the nuclear extracts, 20 μg was separated by SDS-PAGE (12.5%) and electrotransferred onto nitrocellulose membranes (Pall Corporation, Pensacola, FL, USA). Loading of equal amounts of protein in each lane was verified by Ponceau S (Carl Roth, Karlsruhe, Germany) staining. After incubating the membranes overnight at 4 °C in blocking solution [Tris-buffered saline with Tween20 (TBS-T) containing 5% BSA], membranes were incubated in TBS-T with primary antibodies against PPARD (rabbit polyclonal anti-PPARD antibody; 1:500; Abcam, Cambridge, UK), PGC-1 (rabbit polyclonal anti-PGC-1 antibody; 1:1000; Millipore, Darmstadt, Germany) and β-actin (mouse monoclonal anti-β-actin antibody; 1:5000; Abcam) as a reference protein for normalization overnight at 4 °C. The membranes were washed, and then incubated in TBS-T with 3% BSA and horseradish peroxidase-conjugated secondary polyclonal anti-mouse-IgG antibody (rabbit polyclonal; 1:10,000; Abcam) or anti-rabbit-IgG antibody (goat polyclonal; 1:10,000; Sigma-Aldrich, Steinheim, Germany) for 2 h at RT. Afterwards, blots were washed three times with TBS-T and developed using enhanced chemiluminescence (Amersham ECL Select Western Blotting Detection Reagent, GE healthcare, Freiburg, Germany). The signal intensities of specific bands were detected with a Bio-Imaging system (Syngene, Cambridge, UK) and quantified using Syngene GeneTools software (nonlinear dynamics; Syngene). For calculation of protein levels, the band intensity of the proteins of interest was normalized by that of β-actin.

### Statistical analysis

Data are shown as mean values with their standard deviations (SD). The data were subjected to ANOVA using the Minitab Statistical Software Rel. 13.1 (Minitab, State College, PA, USA). The data of experiment 1 were subjected to 1-factorial ANOVA and those of experiment 2 to 2-factorial ANOVA with classification factors being carnitine (CARN), pivalate (PIV) and the interaction of both factors (CARN x PIV). For statistically significant F values, individual means of the treatment groups were compared by Fisher’s multiple range test. Means were considered significantly different for *P* < 0.05. The number of animals used in each group for detecting a statistically significant difference among groups assuming a hypothesized effect size of 1.40, a type I error of 0.05 (two-sided) and a statistical power of 0.95 was calculated by G*Power rel. 3.1.2 [38] taking into account the tissue concentration of total carnitine.

## Results

### Performance parameters of the pigs in experiments 1 and 2

In both experiments, initial and final body weights, daily body weight gain, daily feed intake and feed conversion ratio did not differ between groups (Tables 2 and 3).

**Table 2:** Performance parameters of pigs in experiment 1

	Treatment					ANOVA (*P*-value)
	CON n = 4	CARN *n* = 4	PIV30 *n* = 4	PIV60 *n* = 4	PIV120 n = 4	
Initial body weight	7.71 ± 0.91	7.62 ± 1.02	7.66 ± 0.91	7.35 ± 1.47	7.39 ± 1.59	n.s.
Final body weight (kg)	19.9 ± 1.24	22.3 ± 4.66	19.1 ± 3.51	17.9 ± 5.17	17.9 ± 4.16	n.s.
Daily body weight gain (g)	430 ± 30	520 ± 130	410 ± 90	380 ± 160	380 ± 100	n.s.
Daily feed intake (g)	705 ± 15	545 ± 90	675 ± 115	950 ± 140	740 ± 100	n.s.
Feed conversion ratio (g feed/g gain)	1.71 ± 0.03	1.02 ± 0.40	1.54 ± 0.12	2.36 ± 0.63	1.70 ± 0.55	n.s.

Data are means ± SD. Abbreviations: CON, control group; CARN, carnitine group; PIV30, pivalate 30 mg/kg BW group; PIV60, pivalate 60 mg/kg BW group; PIV120, pivalate 120 mg/kg BW group; n.s., not significant

**Table 3:** Performance parameters of pigs in experiment 2

	Treatment				ANOVA (*P*-value)		
	CON *n* = 12	CARN *n* = 12	PIV n = 12	CARN + PIV n = 12	CARN	PIV	Interaction
Initial body weight	8.05 ± 1.73	7.99 ± 1.22	7.94 ± 0.99	8.00 ± 1.51	n.s.	n.s.	n.s.
Final body weight (kg)	18.7 ± 3.00	21.4 ± 3.12	20.8 ± 2.71	21.3 ± 4.68	n.s.	n.s.	n.s.
Daily body weight gain (g)	382 ± 70	472 ± 91	449 ± 76	460 ± 123	n.s.	n.s.	n.s.
Daily feed intake (g)	739 ± 118	756 ± 184	748 ± 117	841 ± 86	n.s.	n.s.	n.s.
Feed conversion ratio (g feed/g gain)	1.96 ± 0.26	1.66 ± 0.49	1.67 ± 0.13	1.84 ± 0.18	n.s.	n.s.	n.s.

Data are means ± SD. Abbreviations: CON, control group; CARN, carnitine group; PIV, pivalate group; CARN + PIV, carnitine + pivalate group; n.s., not significant

### Concentrations of free carnitine in plasma, liver and LD and BF skeletal muscles of pigs in experiment 1

**Fig. 1:**
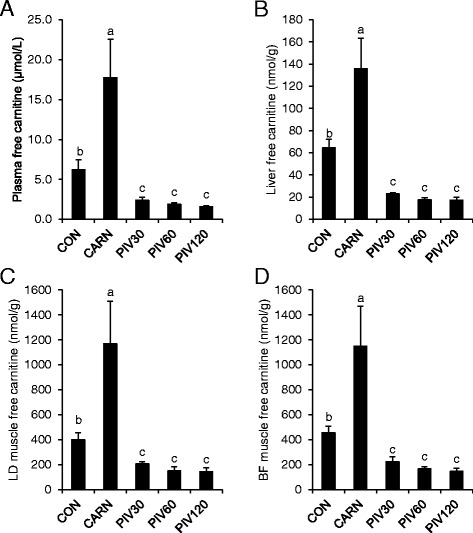
Concentrations of free carnitine in plasma (**a**), liver (**b**), longissimus dorsi (LD) muscle (**c**) and biceps femoris (BF) muscle (**d**) of pigs of groups CON, CARN, PIV30, PIV60 and PIV120 (experiment 1). Bars are means ± SD of n = 4 piglets/group. Bars with different superscript letters differ, *P* < 0.05

The concentrations of free carnitine in all tissues investigated were 2.1–2.9 fold higher in group CARN than in group CON (*P* < 0.05, Fig. 1), but the concentrations of free carnitine were decreased by 45 to 64% in plasma and tissues in the PIV30 group compared to the CON group (*P* < 0.05, Fig. 1). In groups PIV60 and PIV120 the plasma and tissue carnitine concentrations were only slightly lower than in the PIV30 group, but this effect was not significant. Since daily body weight gains were lower and feed conversion ratios were higher in the PIV60 and PIV120 groups than in the PIV30 group, we decided to use 30 mg pivalate/kg BW to induce carnitine deficiency in experiment 2.

### Concentrations of free carnitine and acetyl-carnitine in plasma, liver and LD and BF skeletal muscles of pigs in experiment 2

Concentrations of free carnitine and acetyl-carnitine in plasma, liver and LD and BF skeletal muscles are shown in Table 4. The concentrations of free and acetyl-carnitine in all tissues investigated were highest in group CARN. The concentration of free carnitine in these tissues was 2.0–2.7 fold higher and that of acetyl-carnitine 1.5–3.3 fold higher in group CARN than in group CON (*P* < 0.05). Group PIV had the lowest concentrations of free carnitine and acetyl-carnitine in all these tissues. Concentration of free carnitine in these tissues was 1.9–2.5-fold lower and that of acetyl-carnitine 1.8–2.5 fold lower in group PIV than in group CON (P < 0.05).

**Table 4:** Concentrations of free and acetyl-carnitine in plasma and tissues of pigs in experiment 2

	Treatment				ANOVA (*P*-value)		
	CON n = 12	CARN *n* = 12	PIV n = 12	CARN + PIV n = 12	CARN	PIV	Interaction
Plasma, μmol/L							
free	5.9 ± 0.9^b^	15.8 ± 3.8^a^	2.4 ± 1.0^c^	7.8 ± 2.8^b^	0.001	0.001	0.003
acetyl	0.74 ± 0.14^b^	1.87 ± 0.59^a^	0.35 ± 0.12^c^	0.94 ± 0.31^b^	0.001	0.001	0.009
Liver, nmol/g wet tissue							
free	61.2 ± 12.0^bc^	119.3 ± 23.2^a^	25.9 ± 8.9^c^	74.5 ± 30.7^b^	0.001	0.001	n.s.
acetyl	1.04 ± 0.85^bc^	3.42 ± 1.83^a^	0.42 ± 0.55^c^	1.83 ± 1.47^b^	0.001	0.004	n.s.
LD muscle, nmol/g wet tissue							
free	456 ± 132^c^	1095 ± 225^a^	213 ± 62^d^	609 ± 173^b^	0.001	0.001	0.011
acetyl	296 ± 57^b^	440 ± 83^a^	167 ± 33^c^	303 ± 78^b^	0.001	0.001	n.s.
BF muscle, nmol/g wet tissue							
free	429 ± 93^c^	1034 ± 207^a^	231 ± 64^d^	547 ± 130^b^	0.001	0.001	0.001
acetyl	255 ± 44^b^	459 ± 63^a^	118 ± 34^c^	262 ± 65^b^	0.001	0.001	n.s.

Data are means ± SD. Abbreviations: CON, control group; CARN, carnitine group; PIV, pivalate group; CARN + PIV, carnitine + pivalate group. ^a, b, c, d^Values with different superscript letters differ, *P* < 0.05

The concentrations of free and acetyl-carnitine in plasma and liver did not statistically differ between group CARN + PIV and group CON, but the concentration of free carnitine was 1.2–1.3 fold higher and that of acetyl-carnitine 1.2–1.8 fold higher in group CARN + PIV than in group CON. In LD and BF skeletal muscles, the concentration of free carnitine was 1.3 fold higher in group CARN + PIV than in group CON (*P* < 0.5), whereas the concentration of acetyl-carnitine was similar between group CARN + PIV and group CON.

### Fiber type composition of LD and BF muscles of pigs in experiment 2

**Fig. 2:**
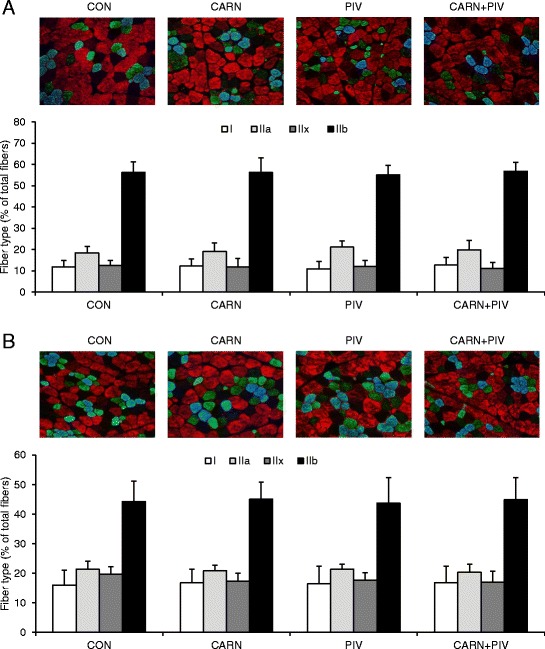
Fiber type composition of longissimus dorsi (**a**) and biceps femoris (**b**) muscle of pigs of the groups CON, CARN, PIV and CARN + PIV (experiment 2). Representative images were obtained from serial muscle cross-sections incubated with a primary antibody cocktail against MHCI (BA-F8), MHC IIb (BF-F3) and all MHC isoforms except MHC IIx (BF-35), followed by incubation with appropriate fluorescent-conjugated secondary antibodies. Images show the four major fiber types, type I (blue), type IIa (green), type IIb (red) and type IIx (unstained). Bars represent fiber type percentages obtained from fiber counting of immunohistochemically-stained muscle cross-sections at 100-fold magnification and are means ± SD of n = 12 piglets/group

As shown in Fig. 2a (LD muscle) and b (BF muscle), immunofluorescence analysis on single muscle cross-sections enabled us to identify the four major fiber types, type I (blue), type IIa (green), type IIb (red) and type IIx (unstained). For both muscles, fiber typing revealed a predominance of fast type II fibers (85–89%), with type IIb being the most abundant followed by type IIa and type IIx. The percentage of slow type I fibers was between 11–13% (LD muscle) and 16–17% (BF muscle). No differences were observed between the four groups in the percentages of type I, type IIa, type IIb and type IIx fibers (Fig. 2a and b).

### Expression of key regulators of muscle fiber composition in LD and BF muscles of pigs in experiment 2

**Fig. 3:**
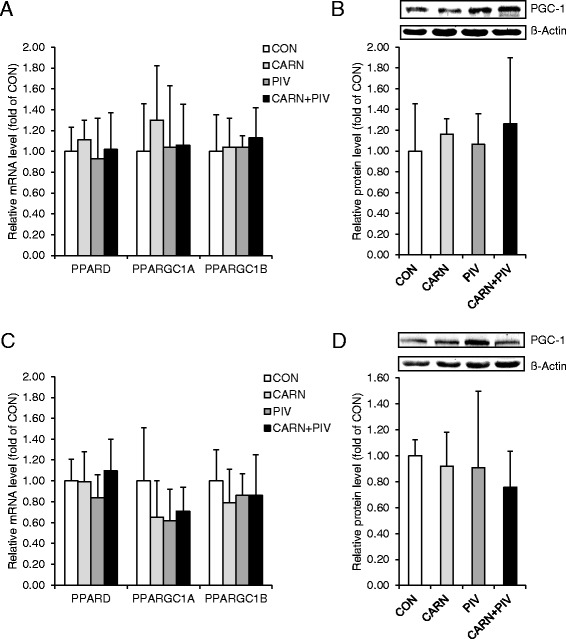
Relative mRNA and/or protein levels of key regulators of muscle fiber composition, peroxisome proliferator-activated receptor δ (PPARδ) and PPARγ coactivators (PGCs) in longissimus dorsi (**a**, **b**) and biceps femoris (**c**, **d**) muscle of pigs of the groups CON, CARN, PIV and CARN + PIV (experiment 2). A, C: Bars represent means ± SD of n = 12 piglets/group and are relative mRNA levels expressed as fold of group CON which was to 1.0. C, D: Bars represent means ± SD of *n* = 6 piglets/group and are relative protein levels expressed as fold of group CON which was to 1.0. One representative immunoblot for PGC-1 and β-actin is shown. Gene symbols and names: PPARD, peroxisome proliferator activated receptor delta; PPARGC1A, PPARG coactivator 1 alpha; PPARGC1B, PPARG coactivator 1 beta

To investigate whether the unaltered fiber type composition is due to the lack of treatment to affect the key regulators of muscle fiber composition, we determined the mRNA levels of PPARD, PPARGC1A and PPARGC1B and protein level of PGC-1α of LD and BF muscles. Figure 3 shows that the mRNA levels of PPARD, PPARGC1A and PPARGC1B in LD (a) and BF (c) muscles did not differ between the four groups of pigs. In addition, the protein level of PGC-1α in LD (b) and BF (d) muscles were not different between groups (Fig. 3).

### Expression of genes related to the metabolic phenotype of LD and BF muscles of pigs in experiment 2

**Fig. 4:**
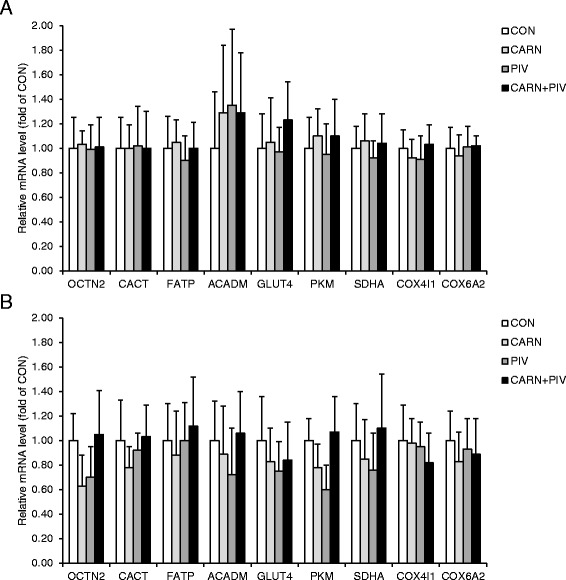
Relative mRNA levels of genes related to the metabolic phenotype in longissimus dorsi (**a**) and biceps femoris (**b**) muscles of pigs of the groups CON, CARN, PIV and CARN + PIV (experiment 2). Bars represent means ± SD of n = 12 piglets/group and are expressed as fold of group CON which was to 1.0. Gene symbols and names: ACADM, acyl-CoA dehydrogenase, C-4 to C-12 straight chain; CACT/SLC25A20, carnitine/acylcarnitine translocase/solute carrier family 25 member 20; COX4I1, cytochrome c oxidase subunit 4I1; COX6A2, cytochrome c oxidase subunit 6A2; FATP/SLC27A1, fatty acid transporter/solute carrier family 27 member 1; GLUT4/SLC2A4, facilitated glucose transporter/solute carrier family 2 member 4; OCTN2/SLC22A5, organic cation/carnitine transporter/solute carrier family 22 member 5; PKM, pyruvate kinase, muscle; SDHA, succinate dehydrogenase complex flavoprotein subunit A

To confirm that the unaltered fiber type composition of skeletal muscles between groups is reflected by an unaltered skeletal muscle metabolic phenotype between groups, the mRNA levels of genes involved in different energy generating pathways, such as fatty acid transport and uptake [fatty acid transporter/solute carrier family 27 member 1 (FATP/SLC27A1)], β-oxidation [acyl-CoA dehydrogenase, C-4 to C-12 straight chain (ACADM)], carnitine shuttle [carnitine/acylcarnitine translocase/solute carrier family 25 member 20 (CACT/SLC25A20)], carnitine uptake [organic cation/carnitine transporter/solute carrier family 22 member 5 (OCTN2/SLC22A5)], TCA cycle [succinate dehydrogenase complex flavoprotein subunit A (SDHA)], oxidative phosphorylation [cytochrome c oxidase subunit (COX4I1), cytochrome c oxidase subunit 6A2 (COX6A2)], glucose uptake [facilitated glucose transporter/solute carrier family 2 member 4 (GLUT4/SLC2A4)] and glycolysis [pyruvate kinase, muscle (PKM)] were determined. In both muscles, the mRNA levels of all these genes did not statistically differ between the four groups of pigs (Fig. 4a, LD muscle; b, BF muscle). However, in BF muscle the mRNA level of OCTN2 was numerically lower in group CARN and group PIV compared to the group CARN + PIV.

## Discussion

The main finding of the present study is that the carnitine status has no effect on the fiber type composition of skeletal muscle in healthy growing pigs. This is based on our observation that the fiber composition of LD and BF muscles was not different between pigs of the CARN group and the PIV group, despite a 5–6-fold difference in plasma and tissue carnitine concentrations. Fiber typing of both muscles was carried out using immunohistochemistry with monoclonal antibodies against MHC I, MHC IIb and all MHC isoforms except MHC IIx. In contrast to the most commonly used method for fiber typing, myosin adenosine triphosphatase (ATPase) histochemistry, which is based on differences between fiber types in the sensitivity of their myosin ATPase activity to pH pre-incubation [39, 40], immunohistochemistry with MHC-specific antibodies allows to clearly delineate the pure (I, IIA, IIB, IIX) and even the hybrid fiber types (I/IIA, IIA/IIX, IIX/IIB) [41]. In the present study, we could detect the pure fiber types in both muscles. In addition, we could realize that the fiber type distribution observed for LD muscle from our pigs is in good agreement with that reported from others using MHC-based fiber typing [42]; similar to our results these authors identified approximately 55% of total fibers as type IIB, 12% as type I and 10–20% as type IIA and IIX in porcine LD muscle. A slight difference between our results and those of Kim et al. [42] is that we did not identify any hybrid fibers, which accounted for 7% of total fibers in LD muscle in the study of Kim et al. [42]. Since we performed fiber typing only in a relatively small area of the muscle sections and the percentage of hybrid fibers is generally low, it is possible that no hybrid fibers were present in our sections by chance. To the best of our knowledge, no data about fiber distribution of porcine BF muscle are available from MHC-based fiber typing. However, our results regarding type I and type IIA fiber percentages in BF muscle are in agreement with fiber distribution data reported previously for porcine superficial BF muscle [43]. In this study a combination of ATPase and SDH staining, which allows to delineate at least type I and type IIA fibers, has been used [43]. In line with the unaffected fiber type composition between the different groups of pigs, we did not observe any differences in the mRNA levels of PPARD, PPARGC1A and PPARGC1B and the protein levels of PPARδ and PGC-1α. All of them are regulators of genes involved in type II to type I fiber transition, mitochondrial biogenesis and oxidative metabolic pathways through activating a variety of nuclear receptors and additional transcription factors, like myocyte enhancer factor 2 (MEF2), nuclear respiratory factor-1 (NRF-1), estrogen-related receptor α (ERRα) and myofiber regulation factors (MRFs) [9–11]. For instance, activation of the MEF2 family of transcription factors stimulates specifically the expression of MHC genes from oxidative fibers [9, 12], while activation of NRF-1 and ERRα causes stimulation of oxidative phosphorylation and mitochondrial biogenesis [44]. Due to these functions, PGCs and PPARδ are typically higher expressed in oxidative type I muscle fibers than in glycolytic type II muscle fibers [10] and mRNA expression PPARs and PGCs correlates with oxidative fiber content in skeletal muscle [45]. In line with this, transgenic pigs with overexpression of the PPARGC1A gene in skeletal muscle have an increased number of type I fibers and pronounced oxidative phenotype [46]. Also in line with the unaffected fiber type composition, no differences were seen in the mRNA levels of genes involved in different energy generating pathways including fatty acid transport and β-oxidation, TCA cycle, oxidative phosphorylation and glycolysis. Thus, our data indicate that supplemental carnitine has no influence on muscle fiber transition and the skeletal muscle metabolic phenotype, which is dependent on the fiber type composition, at least in growing pigs.

Our observation in growing pigs is in contrast to that reported recently in obese Zucker rats [13]. In the latter study L-carnitine supplementation was found to cause an increased percentage of type I fibers, which are typically rich in mitochondria and preferentially use fatty acids for energy production, and a decreased percentage of glycolytic type II skeletal muscle fibers. In line with this, the skeletal muscle of the obese rats fed supplemental L-carnitine exhibited a pronounced oxidative phenotype, as evidenced form increased expression levels of genes involved in fatty acid transport and uptake, β-oxidation, carnitine shuttle, carnitine uptake, TCA cycle and oxidative phosphorylation, compared to obese rats fed no supplemental L-carnitine [13]. Interestingly, the rat study which included also a group of lean rats revealed that the obese state is accompanied by a decrease of type I and an increase of type II fiber percentages in skeletal muscle and an impairment of carnitine status, − effects which are reversed to normal by supplementation of L-carnitine. The L-carnitine-induced type II to type I fiber transition in obese rats has been explained by the observed up-regulation of the abovementioned critical regulators of type II to type I fiber transition, PGC-1α and PPARδ. Similarly, L-carnitine supplementation was reported to increase expression of PGC-1α and PPARδ in rodent models of unloading [5], and genetic and diet-induced obesity and diabetes [47]. Since in the present study the carnitine status did not affect the skeletal muscle phenotype of apparently healthy pigs, it is possible that supplemental L-carnitine is only effective in counteracting the change of muscle fiber distribution induced by pathological conditions (obesity, diabetes, unloading). Nevertheless, we cannot exclude the possibility that the lack of effect of carnitine status in pigs is a species-specific phenomenon which warrants further investigations.

With regard to the situation in human, it has to be mentioned that the dose of supplemental L-carnitine used in this study (20 mg/kg body weight) is roughly comparable to that of a 80 kg person receiving 2 g supplemental L-carnitine, which is a typical oral L-carnitine dose used in clinical trials with healthy and ill subjects (see reviews by [48, 49]). In addition, the dose is of relevance for athletes regularly consuming carnitine supplements, which are advocated to improve exercise performance through enhancing muscle fatty acid oxidation and decreasing reliance on endogenous carbohydrate stores, despite the fact that most of the available clinical studies failed to demonstrate such an effect [26, 48]. One important reason explaining the failure of L-carnitine supplementation to alter muscle fuel metabolism and to improve exercise performance in most clinical studies is probably that carnitine ingestion does not increase muscle free and/or total carnitine content, at least in humans [26–29]. Regarding published effects of supplemental L-carnitine on muscle fiber composition in humans, it has been reported in two independent studies that the fiber composition of vastus lateralis muscle, a muscle with a balanced type I and type II fiber distribution, is not changed by L-carnitine supplementation (either 4 g/day for 6 weeks [50] or 2 × 2 g/day for three month [29]). Thus, the observations in both humans and pigs suggest that supplemental L-carnitine has no effect on muscle fiber composition, despite the fact that the fiber type composition of muscles considered in our pig study differs from that of vastus lateralis muscle. Our assumption is based on the observation that L-carnitine supplementation failed to alter muscle fiber composition in pigs, even though muscle carnitine concentration was strongly increased by the supplemental L-carnitine. The latter observation is in line with previous studies in young pigs [21, 22]. In the study of Fischer et al. [21] it was shown that the concentration free and total carnitine in muscle (LD muscle and semimembranosus muscle) as well as in plasma, heart, liver and kidney linearly increased with increasing dietary L-carnitine supply (0, 25, 50, 100, 200, 500 and 1000 mg/kg diet) during a 20 days feeding period. At the highest dose of 1000 mg L-carnitine/kg diet, muscle total carnitine concentration of pigs was 5-fold higher than in non-supplemented pigs [21]. The reason for the lack of increase of muscle carnitine concentration of humans in response to supplemental L-carnitine is unclear. It has been proposed that species-differences in the bioavailability of supplemental L-carnitine may account for this. While in humans the bioavailability of high oral L-carnitine doses of 1 to 6 g was found to be less than 20% [51–53], in pigs a very high intestinal carnitine absorption rate of >90% has been reported even at a high supplementation level of 1000 mg L-carnitine/kg diet [21]. Similar as in humans, studies in rats demonstrated that high doses of supplemental L-carnitine cause either no [23] or a markedly lower increase of muscle carnitine concentration compared to pigs; a study from our own group with healthy rats demonstrated that feeding a high dose of supplemental L-carnitine (5000 mg/kg diet) for three weeks increased muscle free and total carnitine concentration only by about 50% [25]. The weaker response of the rat compared to the pig may indeed be explained by a lower intestinal absorption rate of carnitine which was determined by the indicator method to be 38% [25]. However, considering that intravenous L-carnitine infusion in humans also failed to impact on muscle carnitine concentration suggests that species differences in the intestinal carnitine absorption rate alone cannot explain the different response of pig, human and rat muscle to supplemental L-carnitine. Based on this, it is much more likely that muscle carnitine transport is the limiting factor to muscle carnitine accumulation. Since carnitine is transported into skeletal muscle against a considerable concentration gradient of greater than 100-fold via a saturable, sodium-dependent, high affinity, active transport process [54], which is catalyzed by the novel organic cation transporter 2 (OCTN2) [55], it appears possible that species differences exist in the expression level of OCTN2 in skeletal muscle. Despite the renal carnitine reabsorption, which is dependent on OCTN2 transport activity in the kidney, is known to be generally high across different mammals, it cannot be excluded that differences in the OCTN2-mediated carnitine reabsorption rate between humans or rats and pigs may be causative for higher renal carnitine losses in humans or rats compared to pigs. This however has to be clarified in future studies. In addition, future studies have to clarify whether long-term supplementation of carnitine in pigs is able to cause fiber switching.

## Conclusion

The present study clearly shows that significant changes in the systemic carnitine status and the muscle carnitine concentration induced by either supplementing L-carnitine or administering pivalate have no impact on the contractile and metabolic phenotype of skeletal muscles containing predominantly type II muscle fibers in pigs. Given that recent studies reported that supplemental L-carnitine has no effect on muscle fiber composition in humans, it can be concluded that pigs and humans behave similar with regard to this phenotypic trait, despite both species differ largely with regard to their ability to accumulate dietary carnitine in muscle.

## Abbreviations

ACADM: Acyl-CoA dehydrogenase, C-4 to C-12 straight chain
BF: Biceps femoris
CACT/SLC25A20: Carnitine/acylcarnitine translocase/solute carrier family 25 member 20
COX4I1: Cytochrome c oxidase subunit
COX6A2: Cytochrome c oxidase subunit 6A2
FATP/SLC27A1: Fatty acid transporter/solute carrier family 27 member 1
GLUT4/SLC2A4: Facilitated glucose transporter/solute carrier family 2 member 4
LD: Longissimus dorsi
MHC: Myosin heavy chain
OCTN2/SLC22A5: Organic cation/carnitine transporter/solute carrier family 22 member 5
PGC-1α: PPARγ coactivator-1α
PKM: Pyruvate kinase, muscle
PPARδ: Peroxisome proliferator-activated receptor δ
SDHA: Succinate dehydrogenase complex flavoprotein subunit A
